# Supplementary material for: The Architectural Layout of Long-Term Care Units: Relationships between Support for Residents’ Well-Being and for Caregivers’ Burnout and Resilience
Source: Int J Environ Res Public Health. 2024 Apr 30;21(5):575. doi: 10.3390/ijerph21050575 (PMC11120887; doi:10.3390/ijerph21050575)
Supplement: Supplementary file 1 [file ijerph-21-00575-s001.zip › ijerph-2842553-supplementary.pdf]

Supplementary Table A1 – *The Psycho-Spatial Evaluation Tool (PSET) - The Twenty-Eight Variables and Measurement Methods*

Variables		Unit	Type of correlation (negative; positive)				
			Physical well-being		Social well-being		
			Comfort	Stimulation	Status	Behavioral confirmation	Affection
Measured from the plan	Number of dedicated public activity rooms (day room or dining rooms)	Numerical	0	1	0	0	1
	Number of internal places that can be used for social interaction	Numerical	0	0	0	0	1
	Number of external places that can be used for social interaction	Numerical	0	0	0	0	1
	Perceived area of dedicated public activity rooms per person	M^2/person	0	-1	0	0	0
	Total unit area- per person	M^2/person	1	0	0	0	0
	Added distance from bedrooms to dedicated public activity room per bedroom	M/bedroom	1	-1	1	0	0
	Added distance from bedrooms to staff base per bedroom	M/bedroom	-1	0	0	-1	-1
	Maximum distance from the staff base to the dedicated public activity areas	Meters	0	0	0	0	-1
	Added distance from the staff base to all support rooms	Meters	0	0	0	-1	-1
	Added distance from the bedrooms to the kitchen per room	M/bedroom	1	-1	0	0	0
	Distance between room with malodor and the central corridor	Meters	1	0	0	0	0
	Minimum distance between room with malodors and main entrance	Meters	0	0	0	0	1
	The longest visual distance in the unit	Meters	0	-1	1	0	0
	Percentage of parallel bedroom doors	Meters	-1	0	0	0	0
	The type of staff base (protrusive, semi-protrusive, obtrusive)	Numerical	0	1	0	1	0
	Penetration experience - the designation of the room located on the route from the main entrance to the dedicated public activity rooms	Numerical	0	0	0	0	-1
	Penetration experience - the designation of the room located on the route from the main entrance to the staff base	Numerical	0	0	0	0	-1
Calculated with Space Syntax	*Integration* - of dedicated public activity rooms	Numerical	0	1	0	1	0
	I*ntegration* - of the staff base	Numerical	0	0	0	1	1
	C*hoice* - in dedicated public activity rooms	Numerical	0	-1	0	0	0
	C*hoice* - in corridors or areas adjacent to the dedicated public activity rooms	Numerical	0	-1	0	0	0
	Intelligibility - levels of orientation	Numerical	0	1	0	0	0
	Visibility - the visibility from and to the staff base	Numerical	1	1	0	1	1
	Visibility - from and to the staff base to the dedicated public activity rooms	Numerical	0	0	0	1	0
	Visibility - the visibility from and to the dedicated public activity rooms	Numerical	0	-1	0	0	0
	Visibility - the visibility to and from the bedroom doors	Numerical	-1	0	1	0	0
	Visibility - to and from the main entrance to the dedicated public activity rooms	Numerical	0	0	0	0	-1
	Visibility - to and from the main entrance to the bedrooms (percentage of the floor area)	Numerical	-1	0	-1	0	0

M^2/person = square meters per number of residents in the unit

M/bedroom = added distance (measured in meters) from bedroom door per number of bedrooms

Supplementary Table A2 - *Regression Coefficients for All Prediction Analyses*

Type of unit	The unit caregivers' burnout and resilience indexes	The architectural layouts' level of support for the residents' five well-being needs					# Architectural layout support for the residents' well-being needs(out of the 5 PSET variables)		# Architectural layout variables (out of the 28 PSET variables)		# Background data (out of 10 background variables)	
		Comfort	Stimulation	Status	Behavioral confirmation	Affection	P>0.05	P>0.01	P>0.05	P>0.01	P>0.05	P>0.01
SDU	Burnout	-		-	-	-	-	-	-	-	-	-
	Work environment.	0.04 (+)	0.04 (-)	-	-	-	-	2	6	1	1	-
	Occupation	-	-	-	-	-	-	-	1			-
	Workplace support	-	-	-	0.05 (-)	-	-	1	7	1	1	-
	Well-being (health)	-	-	-	-	-	-	-	1			-
	Total survey results	-	-	-	-	-	-	-				-
	Unit-caregiver-resident relationship	-	-	-	-	-	-	-	5	1	1	-
	#Predictors	1	1		1	0	0	3	20	3	3	0
RNU	Burnout	-	-	0.02	0.01	-		2	5	3	2	-
	Work environment.	-	-	0.00	0.00	-	2	-	6	2	1	-
	Occupation	-	-	0.00	0.00	-	2	-	5	2	-	-
	Workplace support	0.02	-	-	0.00	-	2	-	4	13	-	1
	Well-being (health)	-	-	-	0.00	-	-	1	11	9	-	-
	Total survey results	-	-	-	0.00	-	-	1	5	12	-	-
	Unit-caregiver-resident relationship	-	-	-	-	-	-	-	4	-	3	1
	#Predictors	1	0	3	6	0	6	4	40	41	6	2
